# Gut Microbiota and Inflammatory Cytokine Changes in Patients with Ankylosing Spondylitis

**DOI:** 10.1155/2022/1005111

**Published:** 2022-08-19

**Authors:** Bin Liu, Zhenghua Ding, Junhui Xiong, Xing Heng, Huafu Wang, Weihua Chu

**Affiliations:** ^1^Lishui People's Hospital, The Sixth Affiliated Hospital of Wenzhou Medical University, The First Affiliated Hospital of Lishui University, Lishui, China; ^2^School of Life Science and Technology, State Key Laboratory of Natural Medicines, China Pharmaceutical University, Nanjing, China

## Abstract

Ankylosing spondylitis (AS) is a chronic inflammatory disease characterized by sacroiliac joint lesions and spinal ascending involvement. The aim of this work was at investigating the gut microbiota profile and proinflammatory cytokines in AS patients. Gut microbiota of AS patients was clearly different from that of healthy human controls. 16S rRNA sequencing analysis demonstrated a changed microbial diversity in the AS patients, and there was a significant increase in the abundance of Cyanobacteria, Deinococcota, Patescibacteria, Actinobacteriota, and Synergistota at a phyla level increased in AS, while the relative abundance of Acidobacteriota, Bdellovibrionota, Campylobacterota, Chloroflexi, Gemmatimonadota, Myxococcota, Nitrospirota, Proteobacteria, and Verrucomicrobiota declined in AS patients. ELISA results for the markers of inflammation in the AS patients revealed increased concentrations of proinflammatory cytokines such as IL-23, IL-17, and IFN-*γ*. Our findings support the fact that the intestinal microbiota are altered in AS with an inflammatory status, which indicates that gut microbiota should be a potential target for ankylosing spondylitis therapy.

## 1. Introduction

Ankylosing spondylitis (AS) is a chronic, progressive disease, which mainly invades sacroiliac joints, paraspinal soft tissue, spinal process, and peripheral joints and can also present with extraarticular manifestations such as anterior uveitis [1, 2]. Spinal deformity and ankylosis can occur in severe cases. The prevalence rate in China is about 0.29%, the male to female ratio is roughly 2~3 : 1, the peak age of onset is 20~30 years, and it is rare above the age of 40 years and below the age of 8 years [3, 4].

Recent studies have shown that there is a certain relationship between ankylosing spondylitis and gut microbiota [5–7]. The human body is home to trillions of microorganisms, and the number of microbial cells in the human body is equivalent to that of human cells [8]. Among them, a large proportion of microorganisms live in our digestive tract and constitute our intestinal microbiome. The research on the correlation between human health and intestinal microorganisms continues to rise, and the correlation between intestinal microecological imbalance and the occurrence and development of diseases has attracted more and more attention. Gut microbiome imbalance is sure to be involved in the process of many immune-related diseases [9]. As research continues, it has been found that gut microbiota plays an important role in the development of ankylosing spondylitis [10–12]. In this study, we compared the alterations of gut microbiota and inflammatory cytokines in AS and healthy human controls.

## 2. Results

### 2.1. Alterations of Inflammatory Cytokines in AS Patients

**Figure 1:**
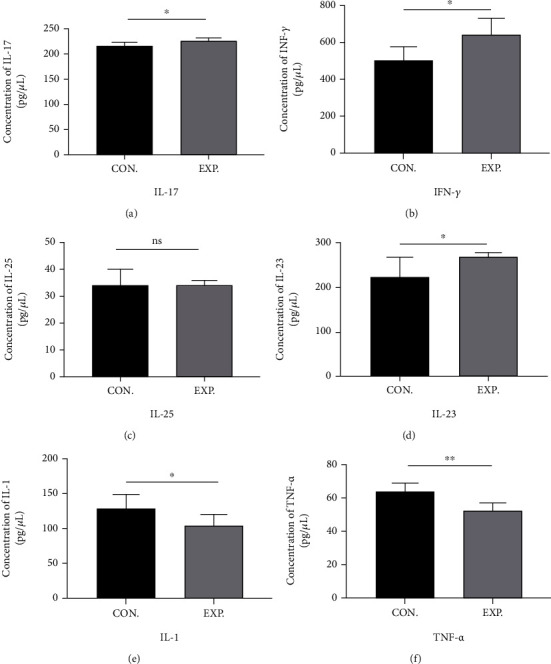
The serum levels of IL-17, IFN-*γ*, IL-25, IL-23, IL-1, and TNF-*α* were determined by ELISA. Data are expressed as means ± standard deviation from 8 person per group. (a) IL-17; (b) IFN-*γ*; (c) IL-25; (d) IL-23; (e) IL-1; (f) TNF-*α*. ^∗∗^*P* < 0.01 and ^∗^*P* < 0.05 vs. control.

As shown in Figure 1, the serum levels of INF-*γ*, IL-17, and IL-23 in the AS patient group were significantly increased when compared with the healthy control. These findings indicate that the levels of proinflammatory factors were increased in AS patients. However, the concentrations of TNF-*α* and IL-1 were significantly reduced compared to those of the healthy control (HC) group. No significant change was detected in IL-25 levels between the AS and HC groups.

### 2.2. Analysis of Gut Microbiota

**Figure 2:**
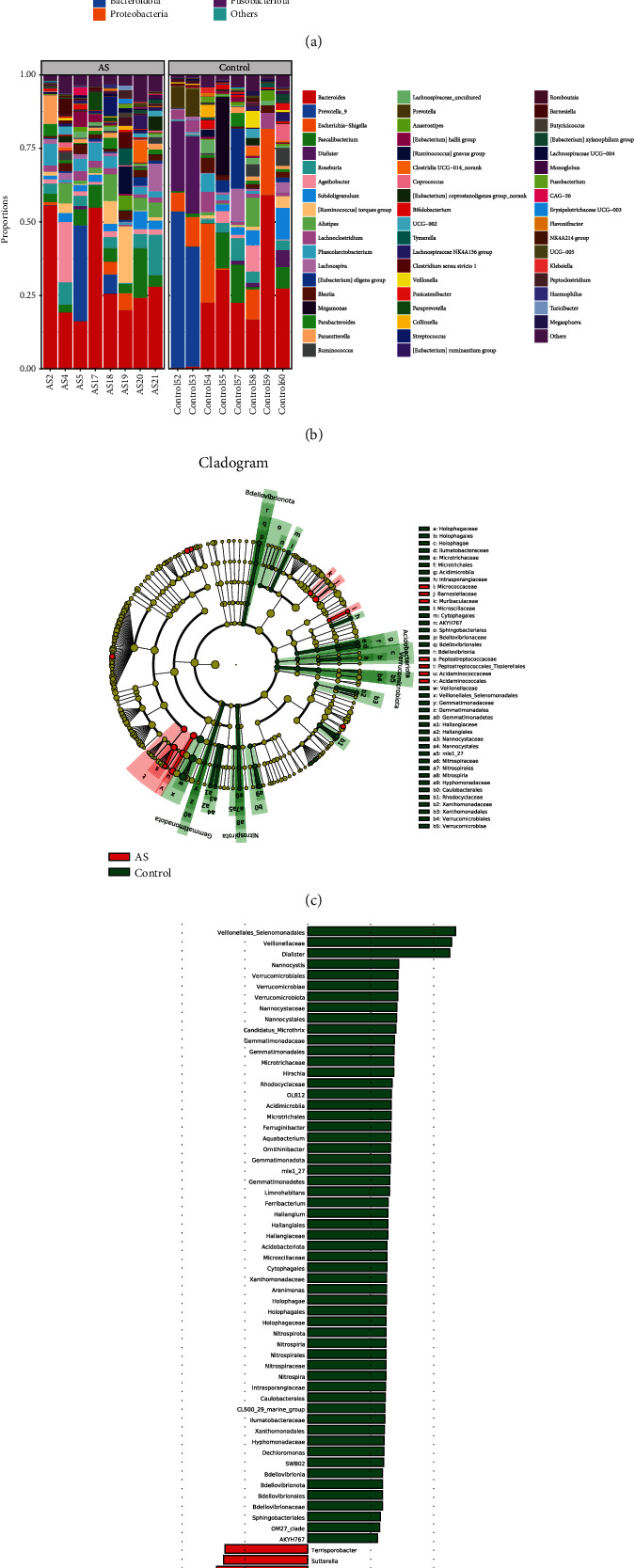
Gut microbial communities are significantly different between AS patients and healthy controls at the phylum (a) and genus (b) levels, and LEfSe analysis on the phylogenetic tree in cladogram format (c) and for LDA scores (d).

With high-throughput sequencing, 805215 high-quality reads which were used to construct OTUs were acquired after filtrating and merging from all samples. At 97% similarity, the amplicons were clustered into 1295 OTUs. By the number of the observed genera, alpha diversity was shown to significantly differ between AS and HCs. The relative abundance of Cyanobacteria, Deinococcota, Patescibacteria, Actinobacteriota, and Synergistota at the phyla level increased in AS, while the relative abundance of Acidobacteriota, Bdellovibrionota, Campylobacterota, Chloroflexi, Gemmatimonadota, Myxococcota, Nitrospirota Proteobacteria, and Verrucomicrobiota declined statistically (*P* < 0.05) (Figure 2(a)). At the genus level, AS patients had an increased relative abundance of *Prevotella_9*, *Alistipes*, *Lachnospiraceae*, *Parabacteroides*, and Ruminococcus in AS (*P* < 0.05). However, the relative abundance of *Dialister*, *Bifidobacterium*, *Veillonella*, *Anaerostipes*, and *Escherichia-Shigella* had declined levels in AS (*P* < 0.05) (Figure 2(b)). In the results from the species level, the community differences between AS and HC groups were analyzed with the level of phylum and genus classification by using the linear discriminant analysis effect size (LEfSe) and linear discriminant analysis (LDA) (Figures 2(c) and 2(d)).

## 3. Discussion

Gut microbiota promotes the development and progression of AS through mechanisms such as increased intestinal permeability and intestinal mucosal immunity. Patients with AS have a unique gut microbiota pattern that may activate autoimmunity. Proinflammatory cytokines such as IL-23, IL-17, IL-10, IFN-*γ*, IL-6, and TNF-*α* are important in the progression of AS. The IL-23/IL-17 immune axis has been shown to be an important factor in the immunopathogenesis of AS [13]. In this study, we found that the levels of IL-23, IL-17, and IFN-*γ* increased significantly in AS patients, while the levels of IL-1 and TNF-*α* decreased, and there was no significant change in IL-25 levels. However, our results were not consistent with the results of Sveaas et al. [14], who concluded that the levels of IL-17 and IL-23 were significantly reduced in AS patients. The level of TNF-*α* has been reported to be significantly decreased in patients with AS [15]. Similarly, in AS patients, a significant decrease in the IL-1 level has been reported [16]. Also, the IFN-*γ* level has been reported to be significantly increased in AS patients [17]. There are limited reports concerning IL-25 levels in AS patients, but several studies have reported significantly increased levels of IL-6 in AS patients [18, 19].

It was found that Firmicutes, Bacteroides, Proteobacteria, and Actinobacteria were the four major microbiota at the phylum level in AS patients and the healthy controls and the abundance of Actinobacteria increased but the abundance of Proteobacteria was significantly decreased in AS patients than in controls, along with Acidobacteriota, Bdellovibrionota, Campylobacterota, Chloroflexi, Gemmatimonadota, Myxococcota, Nitrospirota, and Verrucomicrobiota. Our results are partially consistent with the previous study by Wen et al. [20], which concluded that the Actinobacteria was significantly higher and the Verrucomicrobia was lower in AS patients. However, at the genus level, the *Bifidobacterium* and Prevotellaceae including *Prevotella melaninogenica*, *Prevotella copri*, and *Prevotella* sp. C561 had a higher abundance in AS patients. In addition, when compared with HCs, it was found that the gut microbiota of AS patients demonstrated an increase in the abundance of *Lachnospiraceae*, *Ruminococcaceae*, *Rikenellaceae*, *Bacteroidaceae*, and *Porphyromonadaceae* and a decrease in the abundance of *Veillonellaceae* and *Prevotellaceae* [21]. Some previous studies had also demonstrated an increase in *Klebsiella* in patients with AS [22].

Dysbiosis of gut microbiota is closely related to the occurrence and development of AS, and probiotic supplementation and modification of the dietary structure may help alleviate the condition and symptoms of patients. However, current studies still present several issues, such as the lack of large samples and the lack of long-term follow-up controlled trials. The results of these studies vary widely, and the species of harmful and beneficial bacteria are not yet fully defined. The study of intestinal microbiota regulating the immune system of AS patients still needs to be further explored, thus opening up new avenues for the treatment of AS.

## 4. Conclusions

Richness and diversity of gut microbiota in AS patients were compared with those of healthy human controls. The results indicate that gut microbiota might participate in the pathogenesis of AS by modulating the inflammatory cytokines. We found the gut microbiota in patients with AS has a specific alteration displaying an increase in some bacterial species associated with the decrease in others. Our results are consistent with previous reports. This discovery indicates that gut microbiota should be a potential target for ankylosing spondylitis therapy.

## 5. Methods

### 5.1. Study Participants

In this study, a total of 16 participants were recruited including 8 AS patients and 8 healthy patients matched in age and sex. AS patients were diagnosed in Lishui People's Hospital and collected between August 2020 and March 2021. All AS patients met the ACR/EULAR identification criteria for AS [23]. Healthy controls were recruited from health screening centers of Lishui People's Hospital. Each participant provided informed consent, and the research was approved by the institutional ethics committee of Lishui People's Hospital (ethics number: 2021-342).

### 5.2. Sample Collection

Fresh fecal samples were collected from participants using a sterile box and were transported to the laboratory immediately and then stored at −80° C for use. The blood samples were collected and centrifuged (3000 g for 20 min); then, the serum samples were collected for cytokine analysis.

### 5.3. Detection of Serum Cytokines

Enzyme-linked immunosorbent assay (ELISA) kits (Nanjing Jiancheng Institute of Biotechnology, China) were used to analyze the inflammatory cytokines levels (IL-1, IL-17, IL-25, TNF-*α*, and IFN-*γ*). A Bio-Rad microplate reader (Bio-Rad Laboratories, USA) was used to evaluate the optical density at a wavelength according to the instrument manufacture.

### 5.4. Gut Microbiome Analysis

Total fecal samples of microbial DNA was extracted using the E.Z.N.A.® Stool DNA Kit (Omega Bio-Tek, Norcross, GA, U.S.) according to the protocols of the manufacturer. Partial bacteria of the 16S ribosomal RNA gene (V4–V5 region) were amplified by the PCR method. The PCR products were purified and used to quantify. All purified PCR products were mixed. Library preparation, Illumina sequencing, and bioinformatics analysis were conducted as described by Li et al. [24].

### 5.5. Statistical Analysis

SPSS software (version 22.0, IBM SPSS Inc., USA) was employed to analyze the values. Statistical analysis of variations between the groups was performed using Student's *t*-test. *p* < 0.05 indicated statistically significant differences.

## Data Availability

All novel sequences have been deposited at the NCBI SRA (National Center for Biotechnology Information Sequence Read Archive) https://www.ncbi.nlm.nih.gov/sra) under BioProject PRJNA797884. The other data that support the findings of this study are available from the corresponding author upon reasonable request.

## Abbreviations

ACR: American College of Rheumatology
AS: Ankylosing spondylitis
ELISA: Enzyme-linked immunosorbent assay
EULAR: European League Against Rheumatism
HCs: Health controls
*LDA*: Linear discriminant analysis
LEfSe: Linear discriminant analysis effect size
OTUs: Operational taxonomic units.
